# Seasonality of Mass Shooting Events and Deaths by Suicide in the United States, 2014 to 2021

**DOI:** 10.1177/07487304261464399

**Published:** 2026-07-24

**Authors:** Mark É. Czeisler, Charles A. Czeisler

**Affiliations:** 1 Department of Medicine, Brigham and Women’s Hospital, Boston, MA, USA; 2 Department of Medicine, Harvard Medical School, Boston, MA, USA; 3 Turner Institute for Brain and Mental Health and School of Psychological Sciences, Monash University, Melbourne, VIC, Australia; 4 Division of Sleep and Circadian Medicine, Department of Medicine, Mass General Brigham, Boston, MA, USA; 5 Division of Sleep Medicine, Harvard Medical School, Boston, MA, USA

**Keywords:** epidemiology, morbidity/mortality, firearm violence, public health, violence prevention

## Abstract

It has been hypothesized that most mass shootings are suicidal acts, necessitating investigation of potentially common elements. We investigated whether US mass shootings and deaths by suicide during 1 January 2014 through 31 October 2021 exhibited significant seasonal variations. Outcomes included temporal patterns of mass shooting events and deaths by suicide and their relationship, as characterized by multivariable linear regression models detrended to isolate seasonality and estimate peak phases and amplitudes. Overall, 3,305 mass shootings and 360,827 deaths by suicide were analyzed. Detrended models revealed significant seasonal variation in mass shootings and deaths by suicide. Mass shootings and deaths by suicide displayed concurrent estimated annual peaks occurring on 3 July and 23 June, respectively. Detrended models estimated that at peak-versus-nadir, seasonality accounted for 613% increased mass shootings and 12% increased deaths by suicide. US mass shooting events and deaths by suicide exhibited strikingly synchronous seasonality, with concurrent mid-summer peaks and mid-winter nadirs. This finding calls for expanded implementation of summertime gun violence and suicide-prevention programs and for equipping treatment centers to respond to increased summertime demands. Further research is needed to investigate potential mechanisms underlying the shared seasonality and the hypothesis that many mass shootings are both homicidal and suicidal, intended as final acts.

## Graphical abstract

**Visual abstract:**
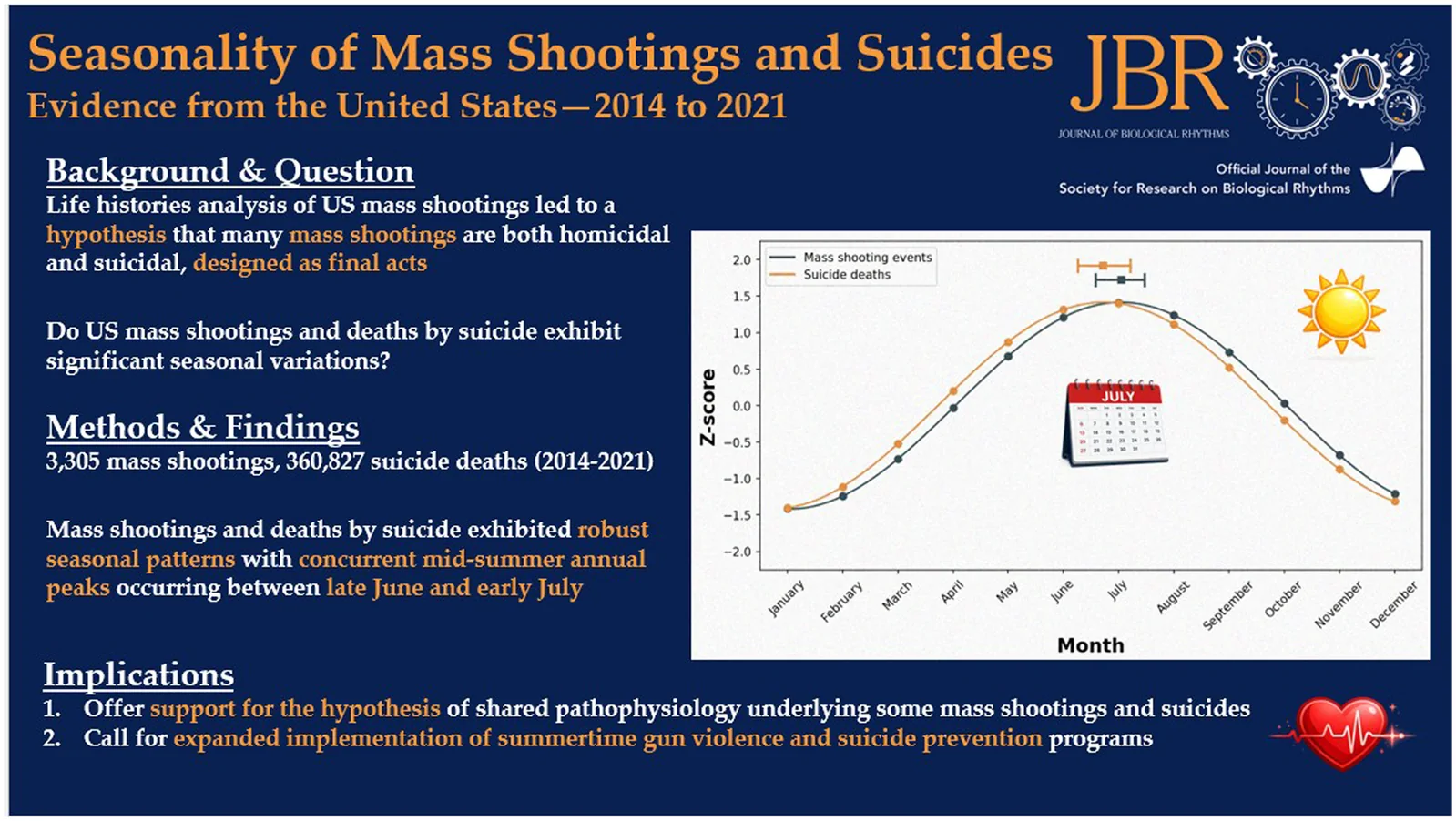

## Introduction

Mass shootings, a gun violence act with markedly increasing incidence in the United States (Peña and Jena, 2021), cause injuries and deaths, often including the shooter. Suicides, a leading cause of US deaths, involve firearms in one-half of cases (Centers for Disease Control and Prevention, 2026). Deaths by suicide are the leading cause of gun deaths, with suicide attempts involving guns resulting in death over 90% of the time (Bauchner et al., 2017). Precipitating circumstances of both mass shootings and deaths by suicide are complex and incompletely understood. However, analysis of US mass shootings that occurred between 1966 and 2021 led to the hypothesis that many mass shootings are both homicidal and suicidal, designed as final acts (Peterson and Densley, 2022).

Since the winter nadir of suicide deaths was first reported nearly 2 centuries ago, this phenomenon has puzzled researchers (Kevan, 1980). Depression is a major risk factor for suicide ideation, attempts, and deaths (Ribeiro et al., 2018), yet depression exhibits robust winter-peaking seasonal variation (Patten et al., 2017), when suicide deaths are paradoxically least common (Kevan, 1980). Interestingly, high-lethality suicide attempts (Aguglia et al., 2019) and suicide deaths by violent methods (Preti and Miotto, 1998) display high-amplitude seasonal variations peaking in summer and spring, respectively, whereas low-lethality suicide attempts and nonviolent suicide deaths do not vary seasonally.

Records observing seasonal variation in mood date to around 400 BC by scholars including Hippocrates (Sher, 2001; Rybakowski, 2024). Insights to pathophysiology of seasonal mood disorders were produced in the late 19th century by American explorer and physician-surgeon Frederick Cook, who described depressive-like symptoms among voyagers and Inuit peoples that he hypothesized resulted from insufficient sunlight (Sher, 2001). In 1984, Norman Rosenthal and Thomas Wehr introduced the contemporary term seasonal affective disorder, a variant of recurrent depression with predictable seasonal changes, treatable with light therapy (Rosenthal et al., 1984). Approximately 20% of people with major depression experience winter seasonal affective disorder (Rybakowski, 2024; Wirz-Justice et al., 2019), the pathogenesis of which involves blunted circadian expression patterns from shortened photoperiods and phase-delay shifts in winter (Rybakowski, 2024).

In contrast to seasonal affective disorder and depression, less is known about mechanisms underlying seasonal variation in deaths by suicide, which are notoriously difficult to predict (Kessler et al., 2020). Mechanisms proposed to contribute to summer-peaking seasonality in suicide deaths include higher ambient temperature (Bär et al., 2022), longer photoperiod (Aguglia et al., 2020), differing social and occupational activities, and biological factors, with violent suicide correlating with seasonal variation in hormone levels and metabolic and immunologic functions (Maes et al., 1996), such as summertime decreases in nocturnal melatonin (Morera and Abreu, 2006) and increases in androgen levels (Lenz et al., 2019). Increased summertime androgen levels could underlie both Aristotle’s and Galen’s 2000-year-old observation that “human nature that is angry, insolent, or fierce” peaks in summer (Grant, 2002) and modern crime statistics showing that gun violence (Klerman et al., 2024) and violent crimes peak during summer, whereas robberies do not exhibit seasonality (Hood and Amir, 2018). These observations extend to depressive phenotypes; winter-peaking depression generally features increased appetite, weight gain, and hypersomnia, whereas summer-peaking depression features decreased appetite and insomnia (Wehr et al., 1991).

Whereas the seasonality of depression and suicidality are well-characterized, the historic rarity of mass shooting events has precluded more definitive characterization of their temporal pattern. Data on seasonal variations in US mass shootings are derived from a single research letter reporting analysis of 3 years of data (Geoffroy and Amad, 2016). Reported nadirs in mass shootings were observed in January 2013, March 2014, and April 2015, with statistically significant cosine fits for seasonal variation reported in 2013 and 2015, but not in 2014. Regrettably, US mass shooting events have risen considerably in recent years (Peña and Jena, 2021), both enabling and necessitating attention to their temporal distributions.

Mass shooting events and suicide deaths most commonly involve young male adults. Of 194 mass shooting events in the United States during 1966 to 2023, 97% were carried out by males with a median age of 32 years (Peterson et al., 2024). While suicidal ideation and attempts are more prevalent among younger adults and females (Olfson et al., 2017; Crosby et al., 2011), attempt lethality and suicide mortality are higher among middle- and older-aged adults and males (Olfson et al., 2017). Accordingly, the 2023 suicide rate among males was roughly 4 times higher than that among females (age-adjusted rate of 22.7 versus 5.9 per 100,000 population), with peaks in midlife (35 to 44 years) and late life (≥85 years) (Centers for Disease Control and Prevention, 2026).

Given the suggestion of commonalities between the mass shootings and deaths by suicides (Peterson and Densley, 2022) and implications of a potential shared temporal relationship for prevention strategies and treatment policies, we sought to investigate whether US mass shooting events and deaths by suicides exhibit significant seasonal variations and, if so, their temporal relationship.

## Methods

Data between 1 January 2014 and 31 October 2021 were examined. Mass shooting events (defined in accordance with the Gun Violence Archive as shootings in which ≥4 people, excluding the shooter, were killed or injured) were obtained from the Gun Violence Archive (n.d.), a gun violence repository generated from >7,500 law enforcement, media, government, and commercial sources.

Suicide data, identified based on the underlying cause of death using *International Statistical Classification of Diseases and Related Health Problems, Tenth Revision* (ICD-10) codes U03, X60-X84, and Y87.0, were obtained from the National Center for Health Statistics CDC Wide-ranging ONline Data for Epidemiologic Research (WONDER) database (Centers for Disease Control and Prevention, U.S. Department of Health and Human Services, n.d.). ICD-10 codes for deaths by suicide were chosen to align with National Center for Health Statistics definition (Hedegaard et al., 2021) and included deaths from intentional means (e.g., self-poisoning, self-harm). Deaths by suicide between 1 January 2014 and 31 December 2020 were obtained from Current Final Multiple Cause of Death Data (Centers for Disease Control and Prevention, National Center for Health Statistics, 2021). Deaths by suicide between 1 January 2021 and 31 October 2021 were obtained from Provisional Multiple Cause of Death Data (Centers for Disease Control and Prevention, National Center for Health Statistics, 2022). All shootings in the Gun Violence Archive repository that met criteria for mass shootings and all deaths classified as suicides by ICD-10 codes in the CDC WONDER database that occurred during the study interval were included.

Calculations included monthly mean mass shootings and deaths by suicide per standardized-length-month, excluding January through October 2021 to minimize secular trend effects within an incomplete calendar year. Multivariable linear regression models were built across all 94 months with trend, sine, and cosine elements to fit a sine wave with a linear trend, then detrended to isolate seasonality and estimate, with 95% CIs, peak phases and amplitudes. For mass shootings, analysis of variance (ANOVA) tests demonstrated superiority of models with either linear or exponential trends compared with no trend. The ANOVA test between models with linear and exponential trends was not significant. For deaths by suicide, the ANOVA tests demonstrated superiority of the model with a linear trend only compared with no trend. The ANOVA test between no trend and an exponential trend was not significant. Therefore, for both outcomes, multivariable linear regression models were used. Estimated z-scores of events per standardized-length-month were plotted. As an exploratory analysis, year-specific seasonal patterns were evaluated by including interactions between year and harmonic terms, and year-specific sine and cosine coefficients were used to estimate year-specific amplitudes and peak phases.

Calculations were performed in Python (V3.7.8) and R (V4.2.0). Statistical significance was defined as 95% CIs excluding the null value. Institutional review board approval was exempted per the Common Rule (45 CFR §46) because the study used publicly available data.

## Results

During the study interval, 3,305 mass shootings and 360,827 deaths by suicide occurred. Between 2014 and 2020, significantly more mass shootings per standardized-length-month occurred in June and July than in January, February, March, or December (Figure 1). Significantly more deaths by suicide per standardized-length-month occurred in August than in January, February, November, or December.

**Figure 1.**
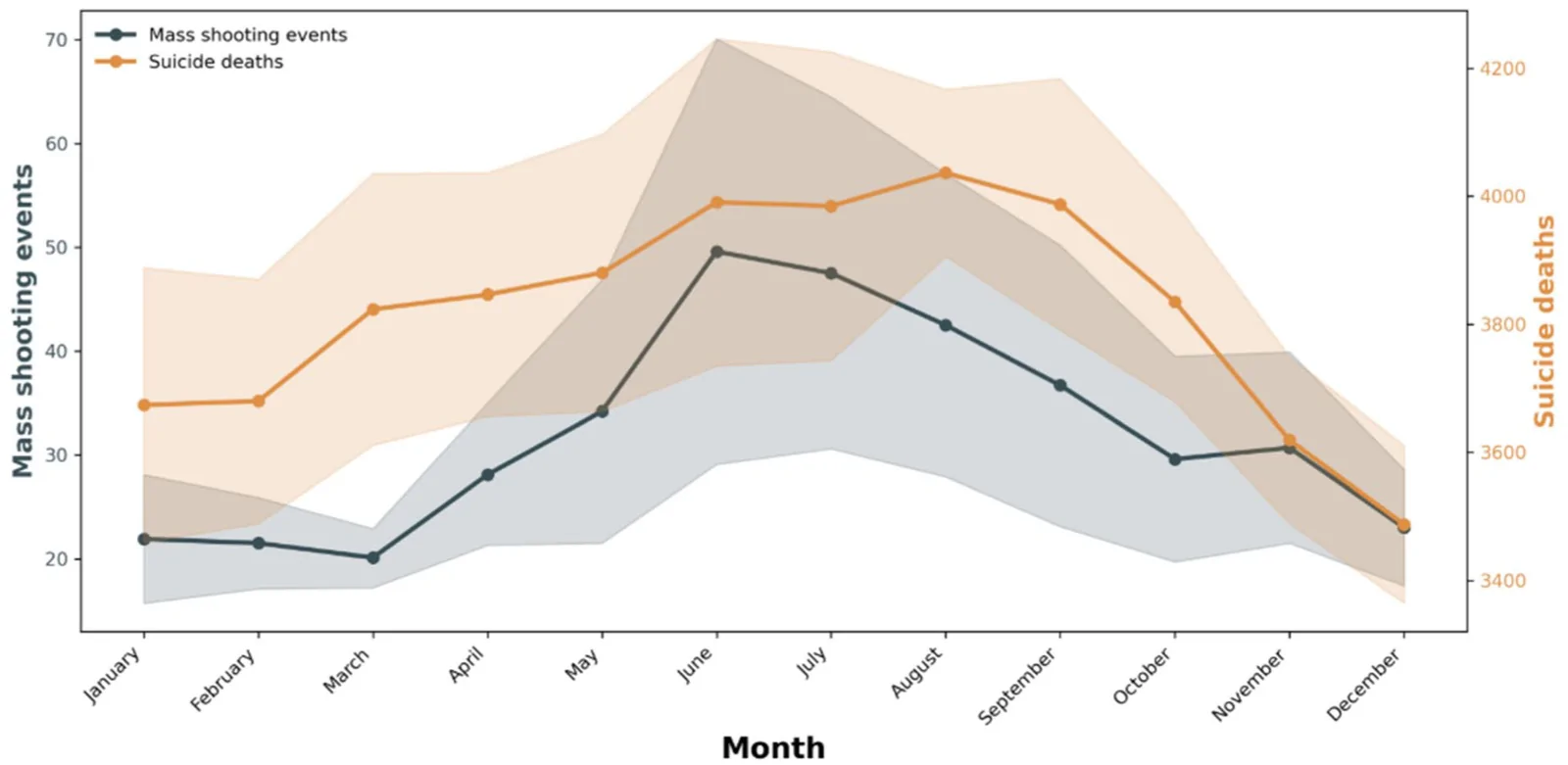
Mass shooting events and suicide deaths per standardized-length-month in the United States, 2014 to 2020. Dots represent mean counts per standardized-length-month for each outcome. Shaded areas represent 95% CIs. The left-sided x-axis presents mass shooting events (gray). The right-sided x-axis presents suicide deaths (orange).

For both mass shooting events and deaths by suicide, the difference-in-means was significantly elevated during June (mean +49.8%, 95% CI [+24.7% to +75.0%]; +4.3% [+1.4% to +7.3%], respectively), July (+46.0% [+28.7% to +63.3%]; +4.2% [+1.2% to +7.2%]), and August (+31.4% [+13.7% to +49.2%]; +5.7% [+3.3% to +8.0%]) and significantly lower during December (−26.5% [−43.0% to −10.0%]; −8.7% [−10.0% to −7.4%], January (−29.3% [−51.3% to −7.3%]; −3.9% [−7.4% to −0.3%]), and February (−30.2% [−47.9% to −12.5%]; −3.7% [−5.8% to −1.6%]). Mass shootings were also lower in March; deathsy by suicide were lower in November.

Detrended models revealed significant seasonal variation in mass shootings and suicide deaths, with amplitudes of 13.4 (10.4 to 16.5) and 203 (153 to 254) events per standardized-length-month, respectively. The temporal relationship of their seasonality reveals concurrent estimated peaks overlapping between 9 June and 16 July (day-of-year 184 [170 to 197] and 174 [160 to 189], respectively) (Figure 2). Detrended models estimated a 613% peak-versus-nadir increase in mass shootings (per standardized-length-month, 31.3 [27.5 to 35.0]; 4.4 [0.6 to 8.2]) and a 12% peak-versus-nadir increase in suicide deaths (3,845 [3,785 to 3,906]; 3,439 [3,377 to 3,501]).

**Figure 2.**
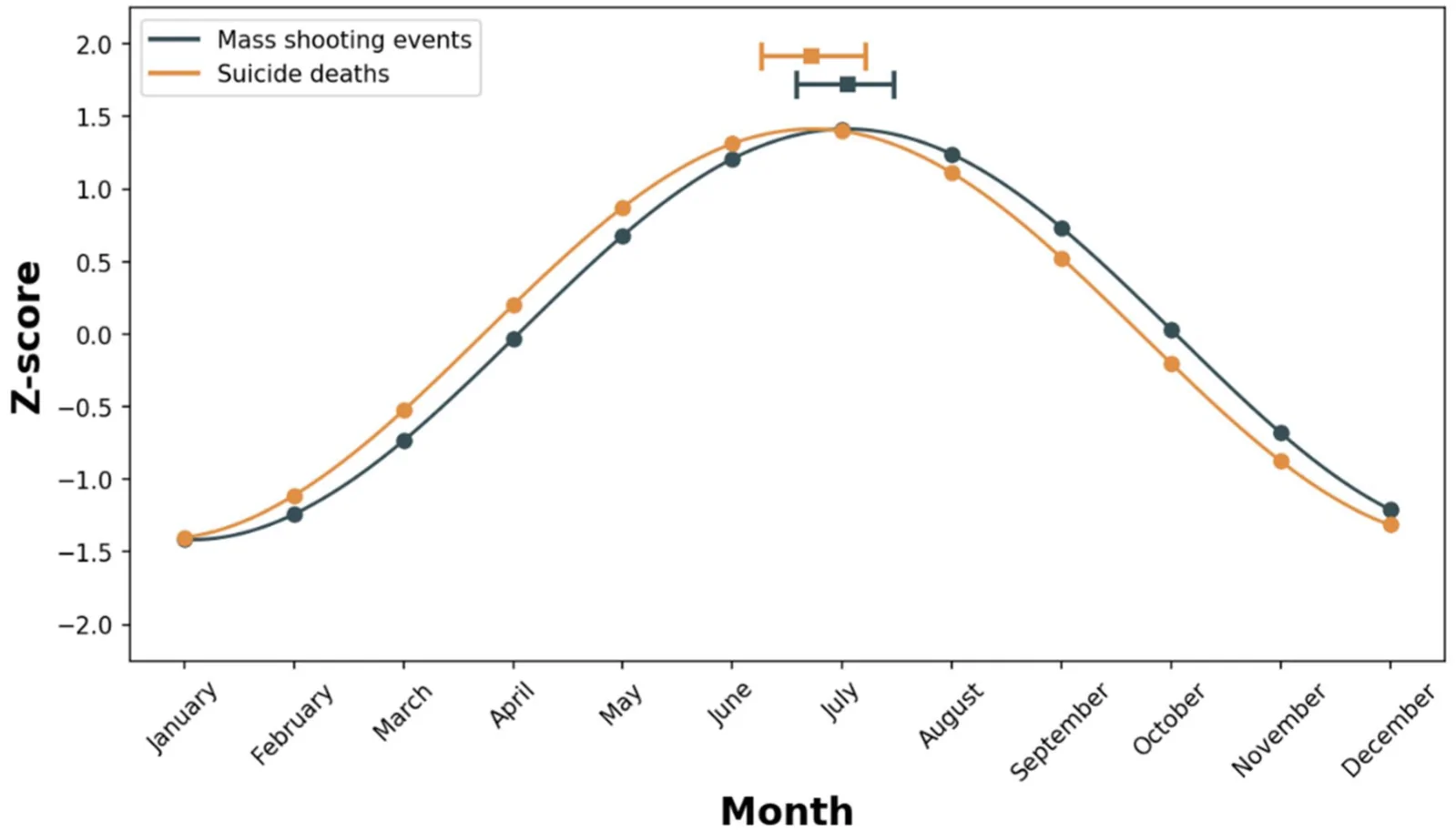
Seasonal component of mass shootings and suicides in the United States, 2014 to 2021. Dots represent monthly estimates for each outcome. Squares represent estimates for the peak phase, with 95% CIs indicated in brackets. For mass shooting events, shown in orange, the peak phase (95% CI) was day-of-year 174 (160 to 189). For deaths by suicide, shown in gray, the peak phase (95% CI) was day-of-year 184 (170 to 197).

Detrended year-specific models revealed that seasonal patterns of mass shootings were largely stable from 2014 through 2019, with amplitudes ranging from 4.9 to 13.4 and the phase of the peak occurring between late May and early July. Consistent with significant year-by-season interactions in 2020 and 2021, seasonal amplitude increased markedly in 2020 (33.3) and remained elevated in 2021 (19.4), with peak phases in early to mid-July. Seasonal amplitudes in suicide deaths were higher during 2014 through 2019 (200.5 to 291.5) than in 2020 (115.1), with partial rebound in 2021 (165.6). The phase of the peak timing spanned late May to early August.

## Discussion

US mass shooting events and deaths by suicide exhibited strikingly synchronous seasonality between 2014 and 2021, with concurrent mid-summer peaks and mid-winter nadirs. This finding calls for expanded implementation of summertime gun violence and suicide-prevention programs and for equipping treatment centers to respond to increased summertime demands. Moreover, alignment of mass shootings with the paradoxically summer-peaking pattern of deaths by suicide and high-lethality suicide attempts, both antiphase with seasonal variations in depression and the summer nadir of low-lethality suicide attempts (Patten et al., 2017; Aguglia et al., 2019), supports the hypothesis that many mass shootings are both homicidal and suicidal, intended as final acts (Peterson and Densley, 2022).

Our revealing of a temporal alignment of US mass shootings and suicide deaths provides epidemiological evidence to complement case-based investigations of the hypothesis that many mass shootings represent externally directed manifestations of suicidality. In 2024, Girgis et al. (2025) identified that 40.5% of individuals who carried out US mass shootings during 2000 to 2019 died by suicide or attempted to do so, nearly doubling the percentage observed during the two preceding decades. Approximately 15% of individuals who attempted suicide during mass shootings had previously expressed suicidal ideation, compared with 3% of those who did not attempt suicide, suggesting that screening for suicidality may allow for intervention.

Our findings come as the public health consequences of mass shootings are amplified by rising frequency. Among 10,000 US adults surveyed in 2024, 7% had witnessed a mass shooting, and nearly 1 in 3 of these witnesses reported having sustained physical injuries (Pyrooz et al., 2025). Exposure to mass shootings has lasting community-level trauma responses, including increased gun (Callcut et al., 2019) and alcohol sales (Buttrick et al., 2025), reinforcing the import of elucidating their underlying mechanisms and enhancing prevention strategies.

Shared predisposing and inciting factors underlying dual-intent (homicide-suicide) mass shootings may partly explain their summertime peak. As early as 1897, French sociologist Émile Durkheim proposed that the summer peak in suicidality reflected a “collective phenomenon” resulting from greater exposure to social demands, expectations, and comparisons amplifying psychological distress among predisposed individuals (Durkheim 1897). Durkheim’s framing has consistencies with the contemporary stress-diathesis model of suicide, which posits that internal (e.g., depression) and external (e.g., acute life stressors) exposures interact with underlying vulnerability (e.g., deficits in executive control) to precipitate suicidal behavior (Mann et al., 1999; van Heeringen, 2012). Beyond suicide, which Durkheim described as self-homicide, stress-diathesis is observed in pathways to violence described in many mass shootings (Peterson and Densley, 2022). A plausible unifying framework is that seasonal variation in activation, impulsivity, irritability, social exposure, and stress-related affective dysregulation may similarly influence the likelihood of both self-directed and other-directed violence among susceptible individuals, with summertime conditions lowering the threshold for the co-expression of violent suicide attempts and mass shootings.

The COVID-19 pandemic served as an acute population-level stressor, with anger among adults peaking during the 2020 summer months (Hagen et al., 2022) before adverse life impacts of the pandemic eased by wintertime, which may partly account for 2020 and, to a lesser degree, 2021 having a higher seasonal amplitude in mass shootings as the incidence rose (Peña and Jena, 2021). The reduced amplitude in suicide deaths corresponded to an unexpected decline in suicide deaths in the early pandemic months (Pirkis et al., 2021) despite increased depression symptoms, suicidal ideation, and suicide attempts (Czeisler et al., 2020; Yan et al., 2023), highlighting the complexity and heterogeneity of suicidality.

Future research priorities include assessing the robustness of these findings across populations and time and probing hypothesized mechanisms. While our study included all deaths by suicide and mass shooting events, as datasets become available and more events accrue over time, subgroup analyses (e.g., young male adults) may yield insights into mechanistically similar events. Given that mass shooting events have been variably defined in the absence of a consensus definition (Bridges et al., 2023), future studies could consider alternative definitions to characterize the generalizability of these findings. Assessment of seasonality across geographic regions would provide insights into potential roles of seasonal exposures such as temperature and photoperiod. If these were contributors, the phase should invert by approximately 6 months among Southern Hemisphere populations, and the amplitude should attenuate where seasonality is weak (e.g., near the equator) and strengthen in high-latitude populations. Global mass shooting research is limited by the relative infrequency of events outside the United States, which accounted for 73% of mass shootings among countries with developed economies during 1998 to 2019 (Silva, 2023).

Regardless of the mechanisms underlying seasonality, these findings support public health and medical community prioritization of summertime efforts to reduce unsafe exposures to firearms, which are required for mass shootings and serve as a highly lethal means of death by suicide. Of note, only 12 of 175 (6.9%) individuals who attempted suicide during mass shootings during 1980 to 2019 survived (Bauchner et al., 2017; Girgis et al., 2025). Epidemiologically informed increased resource allocation in summer months would also address firearm violence in general, which is increased with higher ambient temperature and on weekends and holidays (Klerman et al., 2024; Lyons et al., 2022; Kieltyka et al., 2016; Reeping and Hemenway, 2020).

Study strengths include the use of comprehensive contemporary datasets for mass shootings and suicide deaths and isolation of seasonal components of variation. Limitations include the use of a gun violence repository for mass shootings rather than a national database, which did not yet exist at the time of this analysis, and the use of provisional data for 2021 suicide deaths, which may represent lower-bound estimates due to reporting lags.

In conclusion, mass shootings and deaths by suicide display highly synchronous, summer-peaking seasonality. In parallel with additional mechanistic and epidemiologic investigation into potential commonalities, this finding should promote policies that enhance resources for summertime mental health crisis management and firearm violence prevention.
